# Whole-Genome Sequence of *Lactococcus lactis* Subsp. *lactis* LL16 Confirms Safety, Probiotic Potential, and Reveals Functional Traits

**DOI:** 10.3390/microorganisms11041034

**Published:** 2023-04-15

**Authors:** Justina Mileriene, Jurgita Aksomaitiene, Kristina Kondrotiene, Tora Asledottir, Gerd Elisabeth Vegarud, Loreta Serniene, Mindaugas Malakauskas

**Affiliations:** 1 Veterinary Academy, Department of Food Safety and Quality, Lithuanian University of Health Sciences, Tilžės Str. 18, LT-47181 Kaunas, Lithuania; justina.mileriene@lsmu.lt (J.M.);; 2 Faculty of Chemistry, Biotechnology and Food Science, Norwegian University of Life Sciences, 1433 Ås, Norway

**Keywords:** *Lactococcus lactis*, whole-genome sequencing, probiotic properties, GABA, functional food

## Abstract

Safety is the most important criteria of any substance or microorganism applied in the food industry. The whole-genome sequencing (WGS) of an indigenous dairy isolate LL16 confirmed it to be *Lactococcus lactis* subsp. *lactis* with genome size 2,589,406 bp, 35.4% GC content, 246 subsystems, and 1 plasmid (repUS4). The Nextera XT library preparation kit was used to generate the DNA libraries, and the sequencing was carried out on an Illumina MiSeq platform. In silico analysis of *L. lactis* LL16 strain revealed non-pathogenicity and the absence of genes involved in transferable antimicrobial resistances, virulence, and formation of biogenic amines. One region in the *L. lactis* LL16 genome was identified as type III polyketide synthases (T3PKS) to produce putative bacteriocins lactococcin B, and enterolysin A. The probiotic and functional potential of *L. lactis* LL16 was investigated by the presence of genes involved in adhesion and colonization of the host’s intestines and tolerance to acid and bile, production of enzymes, amino acids, and B-group vitamins. Genes encoding the production of neurotransmitters serotonin and gamma-aminobutyric acid (GABA) were detected; however, *L. lactis* LL16 was able to produce only GABA during milk fermentation. These findings demonstrate a variety of positive features that support the use of *L. lactis* LL16 in the dairy sector as a functional strain with probiotic and GABA-producing properties.

## 1. Introduction

For decades, probiotics have been widely used in foods and beverages due to their positive effects on human and/or animal health [1], generally by improving the functions of intestinal microbiota [2]. A vast majority of probiotic microorganisms belong to lactic acid bacteria (LAB) species. Since *Lactococcus lactis* strains are a principal component of starter cultures, the utilization of indigenous strains provides an important diversity and may contribute to flavour differences and specific technological, nutritional, and health advantages in the development of food products [2,3]. Newly isolated *Lactococcus lactis* strains are also being intensely studied for their probiotic properties [4].

Safety is the most important criteria of any substance or microorganism applied in the food industry. Currently, the primary method for evaluating the safety and probiotic properties of LAB are still in vitro assays. However, even though LAB are generally recognised as safe (GRAS), the rapid rise in antibiotic resistance genes and virulence factors in microorganisms conveys the need to further investigate newly isolated strains by genomic level [5]. The whole-genome sequencing (WGS) of individual LAB strains can be used to assess its safety by analysing the complete genetic information, which includes potential genes coding antibiotic resistance, virulence, and other health-threatening determinants such as the formation of biogenic amines [6]. Moreover, since LAB can also contribute to food safety by producing organic acids, secondary metabolites and/or bacteriocins [7], the genetic analysis can reveal a more in depth understanding of the production of these antimicrobial substances.

Additionally, genomic data can be used to analyse probiotic and functional properties. A potential probiotic strain is anticipated to possess several desirable traits (tolerance to acids and bile, adherence to mucosal and epithelial surfaces, antimicrobial activity against pathogenic bacteria, and bile salt hydrolase activity) in order to exercise its positive effects [8]. The functional properties of LAB can include resistance to suboptimal temperatures, proteolytic activity, formation of flavour compounds, utilization of sugars, etc. [5]. LAB can produce some bioactive compounds during fermentation, including enzymes, vitamins, conjugated linoleic acid, exopolysaccharides [9], and neuroactive compounds, including gamma-aminobutyric acid (GABA), serotonin, dopamine, and acetylcholine [10], which are relevant for the rapidly expanding functional food industry.

A term called “psychobiotics” has recently been proposed to characterize strains that can produce neuroactive substances such as GABA [11]. GABA is a free amino acid that functions as an essential neurotransmitter in mammalian brain [12]. Moreover, GABA is regarded as a bioactive component in the food industry since it serves a variety of physiological purposes, e.g., improving sleep, decreasing anxiety and depression, promoting muscular growth, and regulating blood pressure [13]. The amount of GABA in food can be increased by using GABA producing LAB strains [14]. GABA is formed under acidic conditions from L-glutamate by glutamic acid decarboxylase (GAD) operon. High level of L-glutamate could be released during milk fermentation and proteolysis, as native caseins contain a high proportion of this amino acid [15].

*Lactococcus lactis* subsp. *lactis* LL16 is a novel indigenous strain isolated from raw bovine milk. A previous in vitro study of this strain has confirmed its safety and shown probiotic potential [16], while other studies [17,18,19] involving cheese matrix showed desirable technological and functional properties, such as the ability to positively impact sensory properties and increase the shelf life of cheese. Therefore, the aim of this study was to investigate the whole-genome sequence (WGS) of *L. lactis* LL16 to confirm its safety and gain deeper insights into the genetic basis of its probiotic and functional properties.

## 2. Materials and Methods

### 2.1. Bacterial Strain and Culture Condition

*L. lactis* LL16 strain was previously isolated from locally sourced raw bovine milk and identified using 16S rDNA by Kondrotiene et al. [20]. *L. lactis* LL16 was cultured in MRS broth (Merck, Germany) at 37 °C for 48 h under anaerobic conditions. For the stock culture, the MRS culture broth was mixed with 20% glycerol and kept at −80 °C.

### 2.2. Antibacterial Activity of L. lactis LL16

The antibacterial activity of *L. lactis* LL16 against food spoilage and pathogenic bacteria including *Listeria monocytogenes* ATCC 35152, *Staphylococcus aureus* ATCC 9144, *Escherichia coli* ATCC 35152, *Pseudomonas aeruginosa* NCTC 6750, *Bacillus cereus* ATCC 11778, *Salmonella enterica* serovar Typhimurium ATCC 13311, *Pseudomonas fluorescens* ATCC 13525 and *Brochothrix thermosphacta* ATCC 11509 was evaluated by the method described previously by Kondrotiene et al. [20] using agar spot method. Antibacterial activity was evaluated by measuring clear inhibition zone diameter (mm) around the colony of the tested *L. lactis* strain.

### 2.3. DNA Extraction and Whole-Genome Sequencing

Genomic DNA extraction was performed using the PureLink Microbiome DNA Purification Kit (Invitrogen, Carlbad, CA, USA) according to the manufacturer’s instructions and finally eluted in 50 µL of sterile Mili-Q water. According to the manufacturer’s instructions, DNA libraries were prepared using the Nextera XT library preparation kit (Illumina, San Diego, CA, USA). The sequencing was performed at the University of Copenhagen’s NGS-MiSeq core laboratory utilizing an Illumina MiSeq technology (Illumina) with 250 bp paired-end read format and an average genome depth of 50X. Assembly tool SPAdes v.3.15.3 [21] was used to assemble the reads de novo. QUAST v.5.2.0 [22] was used to assess the assembly’s quality. Prokka v.1.14.6 [23] was used to annotate the constructed sequences.

### 2.4. Identification and Genomic Comparison

Several methods were used to identify species and subspecies. To generate a more traditional species prediction based on the full 16S rRNA gene sequence, the SpeciesFinder v.2.0 tool (https://cge.cbs.dtu.dk/services/SpeciesFinder/, accessed on 22 November 2022) was used [24]. To confirm the identification, a program KmerFinder v.3.0.2 (https://cge.cbs.dtu.dk/services/KmerFinder/, accessed on 22 December 2022) was used to compare the amount of consecutive k-mers between the studied genome and a database of reference genomes [24].

The list of closest phylogenetic neighbours of tested strain were generated by RAST SEED analysis (see below). Then, the OrthoANI tool (https://www.ezbiocloud.net/tools/orthoani/, accessed on 22 December 2022) [25] was used to measure the overall similarity between two genome sequences.

### 2.5. Annotation of Genes Involved in Food Safety

The acquired antibiotic resistance genes were identified using ResFinder v.4.2 (https://cge.food.dtu.dk/services/ResFinder/, accessed on 23 November 2022) available from the Center for genomic Epidemiology (CGE) [26]. Genes encoding bacterial virulence and pathogenicity factors were analysed using VirulenceFinder v.2.0.3 (https://cge.food.dtu.dk/services/VirulenceFinder/, accessed on 24 November 2022) [27] and PathogenFinder v.1.1 (https://cge.food.dtu.dk/services/PathogenFinder/databases, accessed on 24 November 2022) [28] tools. The bacteriocin mining tool BAGEL4 (http://bagel4.molgenrug.nl/, accessed on 23 November 2022) [29] was used to identify putative bacteriocins. Since secondary metabolites produced by bacteria and fungi are an important source of antimicrobials and other bioactive compounds, antibiotics and the secondary metabolite analysis shell-antiSMASH tool (https://antismash.secondarymetabolites.org, accessed on 30 November 2022) were used to identify secondary metabolites [30]. Enzymes coding biogenic amine formation were analysed with RAST-SEED server (see Section 2.6).

### 2.6. Probiotic and Functional Annotation

The subsystems annotation was obtained using the SEED-based automated annotation system after the data were uploaded to Rapid Annotation using Subsystem Technology (RAST v.2.0) genome server (http://rast.nmpdr.org/, accessed on 23 November 2022) [31]. The SEED is a trustworthy tool for predicting gene functions, metabolic pathways, and other bioinformatic data. It is an online database that combines current genomic data. Probiotic features studied included adhesion and aggregation, vitamin biosynthesis, amino acid metabolism, lactic acid production, enzyme production, stress, and host gastrointestinal tract adaptations. NCBI BLAST was used to validate obtained sequences by comparing them to sequences of reference *Lactococcus lactis* strains (https://blast.ncbi.nlm.nih.gov/Blast.cgi, accessed on 22 November 2022) [32].

The ontology and metabolic pathway analysis were performed using Kyoto Encyclopedia of Genes and Genomes (KEGG) server (https://www.kegg.jp/blastkoala/, accessed on 23 November 2022) [33].

### 2.7. Mobile Genetic Elements (MGE) and CRISPR

Mobile genetic elements (MGE), such as insertion sequences (IS), plasmids, prophages, are segments of DNA that encode enzymes and other proteins which facilitate the movement of genetic material between bacterial chromosomes. The MobileElementFinder (version 1.03) (https://cge.food.dtu.dk/services/MobileElementFinder/ [34], accessed on 24 November 2022) was used to detect mobile genetic elements and their relation to antimicrobial resistance genes and virulence factors. Clustered Regularly Interspaced Short Palindromic Repeats were recognized by CRISPRFinder (https://crispr.i2bc.paris-saclay.fr/Server/, [35]), accessed on 22 November 2022.

### 2.8. Complete Genome Sequence Data Accession Number

The sequence data for *L. lactis* LL16 genome were deposited at GenBank under the accession number JARHUB000000000.

### 2.9. In Vitro Investigation

Effect of *L. lactis* LL16 on lactate and free amino acid (FAA) formation was evaluated in sterile milk matrix after 24 h fermentation at 37 °C. Commercial deep-frozen bulk granules of mesophilic LD-type culture of mixed strains (*L. lactis* subsp. *lactis*/*cremoris*; *Leuconostoc* sp.; *L. lactis* subsp. *lactis* var. *diacetylactis*) (Chr. Hansen) was used as a multi-strain control starter (C) for the standard milk fermentation. The culture mix was stored and prepared following the manufacturer’s instructions. Sterile milk (3.5% fat, 3.2% protein, 4.7% carbohydrates) was distributed into 200 mL vats and in triplicate individually inoculated with commercial (C) and *L. lactis* LL16 strain. Inoculation concentrations of all samples started at 5 log cfu/mL (0 h) and ended at 9 log cfu/mL after 24 h of fermentation at 37 °C.

D and L lactate concentrations in milk samples were evaluated with Megazyme assay kit (Megazyme International, Bray, Ireland) after 24 h fermentation and following the manufacturer’s instructions.

Free amino acids (FAA) in fermented milk samples were determined by high performance liquid chromatography (HPLC) according to the method described by Moe et al. with some modifications [36]. Free FAAs were analysed by adding 5.00 mL of internal standard solution (0.1 M HCl, 0.4 µmol/mL L-norvalin (Sigma, St. Louis, MO, USA)) to 5.00 g of sample. The samples were mixed in a Grant-bio mixer, type PV-1 (Grant-Instruments Ltd., England), following by sonication for 30 min. The samples were centrifuged (Thermo Scientific, Heraeus Multifuge X3R, Bremen, Germany) at 2500× *g* for 40 min at 4 °C. A total of 1 mL of 4% TCA (Sigma) was added to 1 mL of the supernatant, mixed in a Grant-bio mixer, and placed on ice for 30 min. After centrifugation at 15,600× *g* for 5 min at 4 °C, the samples were filtered (0.2 µm cellulose acetate filter, Advantec, Dublin, CA, USA) and stored in a freezer (−20 °C) until analysis.

Before separation, 350 µL of borate buffer (0.4 M, pH 10.2, Agilent Technologies) was added to 50 µL of the sample. Separation of amino acids (AA) was performed using Agilent series 1200 instruments: pump, autosampler, column oven, thermostat, and fluorescence detector (Agilent Technologies, Singapore). The system was driven by Open LAB CDS (Agilent Technologies) software. Derivatization with o-phthalaldehyde/3-mercatopropionic (OPA/MPA, Agilent Technologies) was performed at 5 °C. Then, 5 µL of solution was added to 5 µL of sample and mixed 6 times; the reaction time was set to 0.15 min before injection. An XTerra RP 18 column (150 × 4.6 mm; Waters, Milford, MA, USA) was used for separation of AA. Chromatographic conditions were as follows: solvent A, 30 mmol L^−1^ NaOAc pH 7.20 + 0.25% tetrahydrofuran + 0.1 mol/L titriplex III; solvent B, 100 mmol L^−1^ NaOAc pH 7.20 + 80% acetonitrile + 0.1 mol L^−1^ tritriplex III; flow rate 0.7 mL min^−1^, column temperature 42 °C. The derivatized AA were separated by a stepwise linear gradient from 3.3 to 20.7% B over 13 min, and 20.7 to 30% B over 12 min, and 30 to 100% B over 4 min. Column clean-up with 100% B was required for 7 min. The detector parameters were set to detect the OPA derivates at Excitation 340 nm and Emission 455 nm.

Statistical analysis of the data regarding D/L lactate and FAA production was performed with SPSS statistical package (Chicago, IL, USA, SPSS Inc., SPSS 24). Means were compared using Bonferroni’s multiple range tests, results were significant when *p* < 0.05.

## 3. Results and Discussion

### 3.1. Identification and Closest Phylogenetic Relative Analysis

The specie of LL16 isolate was identified as *Lactococcus lactis* based on the complete sequence of the 16S rRNA gene with species prediction tool SpeciesFinder v.2.0 Additionally, prediction using KmerFinder v.3.0.2 revealed the most likely subspecies identification to be *L. lactis* subsp. *lactis*. In this regard, the strain present in the database with the highest query coverage scores (i.e., the percentage of input query Kmers that match the template) was also *Lactococcus lactis* subsp. *lactis* UC06 (91.64%, accession number NZ_CP015902.1; isolated from dairy [37]). These in silico analyses validated our prior incomplete 16S rRNA gene sequencing results [16], which identified our LL16 strain as *Lactococcus lactis* subsp. *lactis*.

Furthermore, RAST SEED analysis detected the 30 closest phylogenetic relatives of *L. lactis* LL16 (Figure S1), the closest one being *Lactococcus lactis* subsp. *lactis* IL1403-a dairy isolate, closely related to the genus *Streptococcus* and commonly used as a cheese starter [38]. The assessment of genetic relatedness using OrthoANI pairwise comparison revealed 98.73% similarity between *L. lactis* LL16 and IL1403 genomes. These results indicate, that *L. lactis* LL16 strain has genomic similarities with other dairy isolates commonly used as dairy starters.

### 3.2. Genomic Annotation

**Figure 1:**
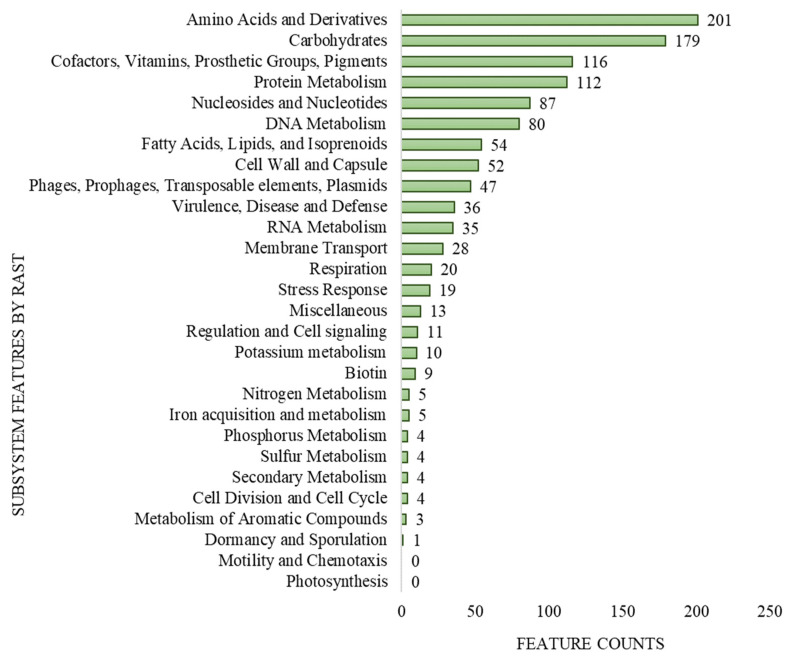
Subsystem coverage and distribution of *L. lactis* LL16 genome by Rapid Annotation using Subsystem Technology (RAST v.2.0) genome server.

The genome size of *L. lactis* LL16 is 2,589,406 base sets, with 35.4% GC content, and 246 *subsystems*. The total number of coding DNA sequences (CDA) and ribonucleotide reductases (RNRs) were 2878 and 63, respectively. The qualities present in the genome of *L. lactis* LL16 were contained on 28% sub-framework and 72% non-subsystem inclusion (Figure 1). These values are similar to those observed for genomes of other *L. lactis* strains [39].

### 3.3. Food Safety Traits

According to The European Food Safety Authority (EFSA), bacterial strains with antibiotic resistance genes should not be used as probiotics for humans or as an additive to animal feeds [40]. Regardless of the fact that LAB are generally recognised as safe (GRAS), every possible LAB probiotic candidate must be tested for transferable antimicrobial resistances since they can still function as a reservoir for antimicrobial resistance genes [6]. ResFinder tool v.4.1 was used to detect genes conferring antibiotic resistance in the *L. lactis* LL16 genome, and none were detected. During previous in vitro studies, *L. lactis* LL16 was found to comply with safety guidelines regarding antibiotic resistance [16].

Virulence genes for Shiga-toxin, *Escherichia coli, Listeria*, and *Enterococcus*; hostimm, exoenzyme, and toxin genes for *Staphylococcus aureus* were also not detected in the search of the VirulenceFinder v.2.0.3 database. Additionally, the PathogenFinder v.1.1 tool identified *L. lactis* LL16 as a non-human pathogen. The chance of being a human pathogen was calculated to be 0.212, with 0 pathogenic and 133 non-pathogenic families matching. These gene search results showed that *L. lactis* LL16 may be safe as a potential probiotic strain without the risk of antibiotic gene transfer. These results are in accordance with the in vitro safety assessment experiments previously performed for *L. lactis* LL16 [16].

Biogenic amines are nitrogenous compounds with low molecular weight that are created in foods by microbial decarboxylation of the source amino acids. Given their potentially harmful neuroactive properties [41], their concentrations in food products should be strictly regulated. Genes related to the formation of biogenic amines, i.e., lysine decarboxylase (EC: 4.1.1.18), ornithine/lysine decarboxylase (EC: 4.1.1.116), arginine decarboxylase (EC: 4.1.1.19), agmatinase (EC: 3.5.3.11), spermidine synthase (EC: 2.5.1.16), arginase (EC: 3.5.3.1), ornithine decarboxylase (EC: 4.1.1.17), histidine decarboxylase (EC: 4.1.1.22), tyrosine decarboxylase (EC: 4.1.1.25), and tryptophan decarboxylase (EC: 4.1.1.28) were not found in the *L. lactis* LL16 genome. Therefore, regarding the biosynthesis of biogenic amines, the use of *L. lactis* LL16 in food products meets the requirements of food safety.

### 3.4. Antimicrobial Activity and Bacteriocin Production

The ability of LAB to synthesize organic acids and/or bacteriocins is frequently related with its antibacterial effectiveness against food spoilage and pathogenic microorganisms [7]. The antimicrobial activity of *L. lactis* LL16 was evaluated both in vitro and in silico. The agar spot assay revealed that *L. lactis* LL16 was able to produce a zone of inhibition (mm) against *L. monocytogenes* ATCC 35152 (8.10 ± 0.01), *S. aureus* ATCC 9144 (8.50 ± 0.01), *E. coli* ATCC 35152 (10.50 ± 0.03), *P. aeruginosa* NCTC 6750 (17.00 ± 0.01), *B. cereus* ATCC 11778 (10.10 ± 0.08), *S.* Typhimurium ATCC 13311 (19.50 ± 0.01), *P. florescens* ATCC 13525 (8.20 ± 0.01), and *B. thermosphacta* ATCC 11509 (10.00 ± 0.03).

**Figure 2:**
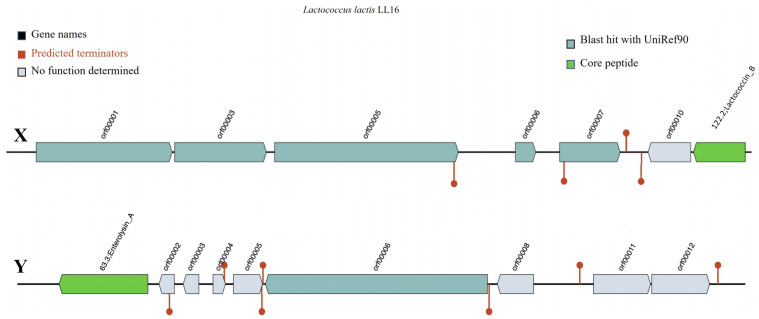
Representation of gene clusters coding the production of putative bacteriocins Lactococcin B (X) and Enterolysin A (Y) in the genome of *L. lactis* LL16 using the online webserver BAGEL v.4.0. Predicted terminators are shown as the maroon line circle ends.

Bacteriocin detection tool BAGEL v.4.0 identified Lactococcin B (LcnB) and enterolysin A (EnlA) as two putative bacteriocinogenic genetic clusters present in the genome of *L. lactis* LL16 (Figure 2). Their levels of identity were 37.50% (E-value = 1.51 × 10^−8^) and 62.90% (E-value = 4.55 × 10^−22^), respectively, thus further investigations are needed. Lactococcin B is a class II bacteriocin with approximately 5 KDa molecular weight that exclusively inhibits the growth of sensitive lactococci. It has a bactericidal effect, but its activity depends on the reduced state of Cys-24 residue [42]. The producer strains have potential applications in the dairy industry as they could be used as starters in cheese-making to mediate the lysis of natural starter strains to accelerate ripening and increase flavour development [42]. Saltaji et al. report [3] that *L. lactis* isolate L14 also harboured a LcnB gene and showed similar antimicrobial activity against *Salmonella* Typhimurium CIP104115 (7.5 ± 0.7), *Staphylococcus aureus* DSMZ13661 (15.5 ± 2.1), *Enterococcus faecalis* CIP103015 (7.5 ± 0.7), and *Listeria innocua* CIP80.11 (18.0 ± 0.0). Zhang et al. [43] report that EnlA in the soluble cellular fraction displayed inhibitory activities against *Bacillus subtilis, Listeria monocytogenes, Listeria innocua,* and *Staphylococcus aureus.*

### 3.5. Secondary Metabolites

**Figure 3:**
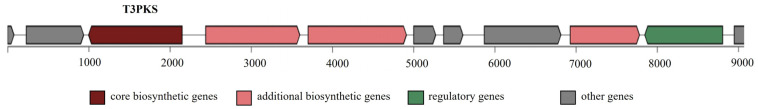
Secondary metabolite-producing region T3PKS in the genome of *L. lactis* LL16 detected with the antiSMASH 5.0 web tool. The arrows indicate the direction of transcription for each gene and the colours indicate gene function.

Bacterial secondary metabolism produces a rich source of bioactive compounds, some of which are of potential pharmaceutical value, e.g., antibiotics, cholesterol-lowering drugs, and antitumor drugs [44]. Here, antiSMASH 5.0 was used to predict secondary metabolic pathways. One region in the *L. lactis* LL16 genome was identified as type III polyketide synthases (T3PKS) to produce bacteriocins involved in food safety (Figure 3). T3PKS is one of the two most abundant biosynthetic gene clusters in all LAB genera [45].

### 3.6. Probiotic Traits

Among the most essential characteristics of a potential probiotic strain is its capacity to adhere to the host’s gastrointestinal system. In this context, the RAST SEED tool discovered genes encoding enolase, fibronectin-binding protein, exopolysaccharides (EPS) biosynthesis, triosephosphate isomerase, sortase A (LPXTG), and ATP synthase ε (epsilon) chain (Table S1). Enolase is a protein that helps the strain attach to the host’s gastrointestinal system, whereas the fibronectin-binding protein enables the bacteria to adhere to the host’s fibronectin [46]. EPS are an alternative class of bio-thickeners widely used in the food industry [47]. The synthesis of EPS by probiotic LAB strains has been proven to significantly improve the texture and rheological properties of fermented foods by inhibiting syneresis, as well as enabling LAB cell adherence to the intestinal mucus of the host [48]. The sortase family protein and LPXTG-motif cell wall anchor domain protein, which are involved in cellular adhesion, are another desired attributes for probiotics in the gut colonization stage [49].

The ability to tolerate gastrointestinal tract conditions, such as low pH and bile salt concentrations, is important for probiotic strains [50]. A previous study by Kondrotiene et al. [16] showed that *L. lactis* LL16 exhibited an important resistance in vitro to gastrointestinal tract conditions (bile salts and acid). Gene annotation confirmed these results since *L. lactis* LL16 had several genes for acid and bile tolerance (Table S1): ATP synthase, including alpha, beta, gamma, epsilon chain and subunit a, b, c, and L-lactate dehydrogenase genes, which contribute to the acid tolerance of the cells as cytoplasmic pH regulates cellular activity related to pH homeostasis. Lactate dehydrogenase is required for ATP production, which increases proton extrusion and enhances acid tolerance in bacteria [50]. Furthermore, glucosamine-6-phosphate deaminase and CTP synthase genes, which are involved in bile salt tolerance, were detected in the genome. *L. lactis* LL16 contains one gene-encoded cyclopropane-fattyacyl-phospholipid synthase which could enhance the synthesis of lipids, such as cyclopropane fatty acid (CFA). The cyclopropane fatty acid defends probiotics from harsh environments, such as exposure to acid, bile salt, or other pollutants [51].

### 3.7. Functional Traits

The capacity of LAB to produce lactate (an end-product of lactic acid fermentation) is also a vital feature since it is known to have an antimicrobial effect. Lactate can be found in both D and L enantiomer forms, depending on whether the genes encoding D-lactate or L-lactate dehydrogenase are present [52]. Both L-lactate and D-lactate dehydrogenases were found in the genome of *L. lactis* LL16 using RAST analysis (Table S1). Therefore, the capacity of *L. lactis* LL16 strain to produce these lactate isomers was evaluated in milk samples after 24 h of fermentation and compared to a commercial starter. The results indicate that *L. lactis* LL16 strain and control produced similar amounts of both L-lactate (0.82 ± 0.01 and 0.86 ± 0.01 mg/100 g, respectively) and D-lactate (0.16 ± 0.01 and 0.18 ± 0.02 mg/100 g, respectively). *L. lactis* utilize the glycolytic pathway involving the phospho-β-galactosidase enzyme to generate L-lactate, while other homofermentative strains that are commonly used in commercial starters (*Lactobacillus helveticus, Streptococcus thermophilus*, etc.) generate D-lactate [53]. High concentrations of D-lactate are harmful to humans and should be avoided since it can have a direct neurotoxic effect even though median lethal doses are quite high (LD_50_ value level per orally poisoned rats is around 4.5 g/kg) [54]. L-lactate is the preferred isomer contributing to flavour profile in food products, especially dairy.

From a technological standpoint, the capacity of the strain to withstand temperature variations are an important aspect since fermented foods are manufactured at varying temperatures and then refrigerated or frozen [55]. The discovery of numerous genes associated with improved resistance to suboptimal temperature settings (such as molecular chaperones *GroES* and *GroEL*, several CSP family proteins, *DnaJ, DnaK*, and *GrpE*) reinforces the technological functionality of *L. lactis* LL16 (Table S1). In our previous study [19], *L. lactis* LL16 strain was able to maintain its viability (>6 log cfu/g) during immobilisation on raisins within various temperature treatments: thermal drying (30 °C/24 h), freeze-drying (−80 °C/24 h), and wet storage (4 °C/24 h).

The previously analysed technological behaviour of *L. lactis* LL16 strain in cheese [17,18,19] is in an agreement with its possession of genomic elements involved in adaptation to the dairy environment, such as the lactose utilization operon (*lacR-ABCDFEGX*), the proteolytic system (*prtC*), and the oligopeptide permease system (*oppDFBCA*) (Table S2).

The production of enzymes can be a desirable attribute in dairy production. With RAST analysis several enzyme coding genes were detected in *L. lactis* LL16 genome, such as α-amylase, lipases, serine protease, DegP/HtrA, and xylanase (Table S1). The ability of lactococci to produce enzymes such as amylase and lipases are both a probiotic and a technological characteristic [46]. Lipases and proteases or proteinases are of fundamental importance in food fermentations and in dairy foods for flavour development.

Vitamin production by LAB has gained the attention of the scientific community. It has been shown that certain foods fermented with LAB contain high levels of B-group vitamins as a result of microbial biosynthesis [56]. Numerous genes responsible for the production of B-group vitamins, such as thiamine (vitamin B_1_), riboflavin (vitamin B_2_), pyridoxin (vitamin B_6_), biotin (vitamin B_7_), and folate (vitamin B_9_), which are known to have a broad range of anti-inflammatory, immunomodulatory, antioxidant, and neuroprotective properties [57], were identified with RAST analysis (Table S1). Interestingly, previous in vitro studies with fresh cheese containing *L. lactis* LL16 strain resulted in products with significant differences in green colour (b* colour coordinate), which could have indicated a higher amount of B-group vitamins [17]. Other studies have investigated the potential uses of riboflavin and folates producing LAB for the biofortification of food, as therapeutics against intestinal pathologies, and to complement anti-inflammatory/anti-neoplastic treatments [58].

### 3.8. Production of Amino Acids, including GABA

The RAST analysis also detected several genetic clusters encoding the metabolism of numerous amino acids (AA), such as threonine, tryptophan, methionine, leucine, lysine, cysteine, histidine, and arginine (Table S1). Proteins and amino acids are fundamental for optimal health and neurological functions. Furthermore, free amino acids (FAAs) are essential precursors for a range of metabolic pathways that result in the production of main flavour compounds found in many cheese varieties [59]. *L. lactis* is used in starter cultures that provide flavour to the product via the proteolytic degradation of the milk protein and subsequent formation of flavour compounds from the AA. In previous studies, *L. lactis* LL16 is known to produce a pleasant aroma during milk fermentation [16] and significantly increase the sensory acceptability of cheese during storage [18,19,60]. It is known that lactococci strains that have been isolated from low-amino acid environments are more dependent on their own biosynthesis of amino acids than industrial starter strains [61]; therefore, wild lactococci exhibit a wide range of amino acid conversion capacities, resulting in diverse aromatic profiles.

Several studies with intestinal epithelial cells showed that some LAB strains could stimulate colonic serotonin synthesis [62]. The genomic analysis confirmed that *L. lactis* LL16 had the tryptophan biosynthetic gene (Table S2), which could produce tryptophan, a precursor that can be used in 5-HT synthesis. Serotonin, also known as 5-HT, is synthesized via a short pathway from L-tryptophan, in which tryptophan hydroxylase and aromatic L-amino acid decarboxylase are involved [50]. The results by Gao et al. [63] indicate that the oral administration of *L. lactis* WHH2078 can alleviate rodent depressive and anxiety-like behaviours, which are associated with the improvement in 5-HT metabolism and modulation of the gut microbiome composition. To confirm the ability of *L. lactis* LL16 to produce tryptophan in a food matrix, further in vitro studies are recommended.

Moreover, the glutamate decarboxylase (GAD) gene cluster (*gadABC*, e-value = 0.0) involved in GABA production was also identified in *L. lactis* LL16 genome (Table S2). GABA, a compound with beneficial effects on human health, is naturally present in many varieties of cheese, although the extent of its accumulation depends on multiple environmental, technological, and metabolic factors. Authors Valenzuela et al. report [64] that out of the tested 262 LAB isolates, the highest amounts of GABA were produced specifically by 16 *L. lactis* subsp. *lactis* strains; all GABA-producing *L. lactis* isolates, except one, harboured GAD genes. The functionality of GAD operon in *L. lactis* LL16 can also be confirmed, since in a previous study [65], significant amounts of GABA were detected in whey cheese samples using this strain.

### 3.9. Insertion Sequences (IS), Plasmids, and CRISPR-Cas

The MobileElementFinder service revealed the presence of three IS (e-value = 0.0), with each open reading frame (ORF) defined as a transposase (Table 1). The presence of CRISPR-Cas systems in the genome of *L. lactis* LL16 was examined with the CRISPRFinder tool. In this regard, a single sequence of CRISPR matched a positive prediction for the existence of CRISPR arrays (Table 1).

One plasmid (repUS4) was found in the *L. lactis* LL16 genome (Table 1) using MobileElementFinder web tool. The plasmid repUS4 (also known as repA_pCI2000) that has been identified contains a similar sequence (99.57%, e-value = 0.0) to the plasmid pCI2000, which is found in the *L. lactis* NCDO 275 genome. The partition mechanism associated with pCI2000 is unique to lactococci and was the first documented example of an active plasmid partitioning system for Gram-positive bacteria [66]. Overall, plasmids of *L. lactis* are involved in essential functions such as bacteriocin production and resistance to antibiotics, along with technological properties such as utilization of citrate, casein, and lactose, stress response and adaptation, exopolysaccharide production, proteolysis, and so on [67]. The results by Kelly et al. [68], analysing 150 dairy starters, show that lactococci genomes averaged 7 plasmids per strain, ranging from 0 to 14, concluding that more plasmids corresponded to a strain with more functional properties.

**Table 1:** Mobile genetic elements (insertion sequences and plasmids) and CRISPR-Cas systems identified with tools MGEFinder and CRISPRFinder in the genome of *L. lactis* LL16.

Analysed Element	*Lactococcus lactis* LL16		
Insertion Sequences	Accession	Lenght (bp)	Reference
IS6(ISS1B)	D63820 ^a^	13,685	[69]
IS6 (ISS1N)	M37395 ^b^	13,879	[69,70]
IS6 (ISLla3)	CP003132 ^c^	1682	[71]
Plasmids	Accession	Identity	
repUS4	AF178424 ^d^	99.57%	[66]
CRISPR-Cas systems	CRISPR spacers	DR *	
	3	23	

^a^*—Lactococcus lactis* subsp. *lactis* bv. diacetylactis B-1 (pTL02); ^b^—*Lactococcus lactis* subsp. *cremoris* SK11 (pSK111); ^c^—*Lactococcus lactis* subsp. *cremoris*; ^d^—*Lactococcus lactis* NCDO 275, plasmid pCI2000; * DR—the number of target base pairs duplicated on insertion.

### 3.10. GABA Production in Fermented Milk

**Figure 4:**
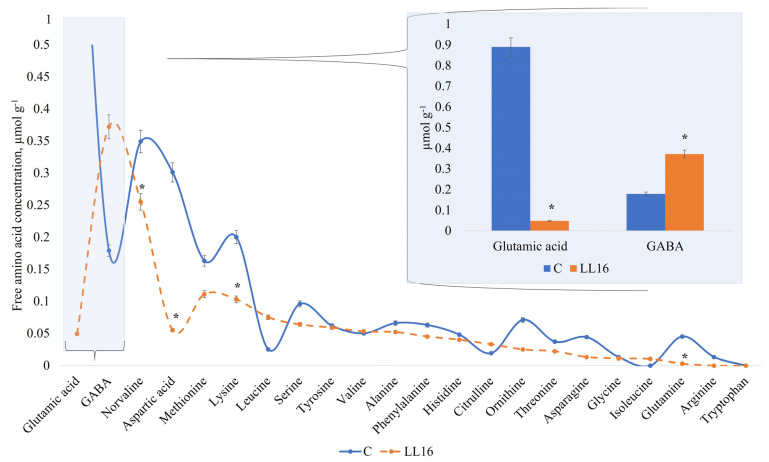
Free amino acid concentration (µmol g^−1^) in an experimental milk matrix with *Lactococcus lactis* strain LL16 and commercial starter (C) after 24 h of fermentation at 37 °C. Statistically significant differences (*p* < 0.05) are indicated by (*).

The profile of free amino acids (FAAs) displayed similar activity in all of the samples for amino acids detected in our study, except for two of them—glutamic acid and GABA (Figure 4). The high amount of GABA in the milk sample fermented with *L. lactis* LL16 points out the ability of this strain to synthesize most of it by converting glutamine into glutamate and then to GABA, referring to the findings of Mazzoli et al. with *Lactococcus lactis* NCDO 2118 [72]. Other authors also reported some *Lactococcus lactis* strains that produced GABA in fermented milk [73] and cheese [74], and that the amount of GABA produced varied among all these tested strains. However, GABA production could be improved by optimizing the culture conditions [75]. Therefore, GABA production by *Lactococcus lactis* LL16 strain could be enhanced by optimizing cultivation or fermentation conditions. Our results show that *L. lactis* LL16 strain produced double the amount (*p* < 0.05) of GABA that was produced in milk fermented with the commercial starter (Figure 4). Thus, further research is needed in order to increase GABA yield by this strain. The results of FAA also indicate that, even though *L. lactis* LL16 strain harbours the tryptophan biosynthetic gene, it did not produce tryptophan during 24 h of milk fermentation.

## 4. Conclusions

The whole-genome sequencing of an indigenous dairy isolate LL16 confirmed it to be *Lactococcus lactis* subsp. *lactis* with genome size 2,589,406 bp, 35.4% GC content, 246 subsystems, and 1 plasmid (repUS4). The safety of *L. lactis* LL16 strain was confirmed with low pathogenicity values and the absence of genes involved in transferable antimicrobial resistances, virulence, and formation of biogenic amines. The in vitro analysis of *L. lactis* LL16 showed antimicrobial activity against *Listeria monocytogenes*, *Staphylococcus aureus*, *Escherichia coli*, *Pseudomonas aeruginosa*, *Bacillus cereus*, *Salmonella* Typhimurium, *Pseudomonas florescens,* and *Brochothrix thermosphacta*. One region in the *L. lactis* LL16 genome was identified as T3PKS to produce putative bacteriocins involved in food safety. These included Lactococcin B and Enterolysin A. The previously identified probiotic potential of *L. lactis* LL16 was further investigated due to the presence of genes encoding adherence to the gastrointestinal tract of the host, acid and bile tolerance, and lactate production. The functional annotation of *L. lactis* LL16 genome revealed genes involved in an increased resistance to suboptimal temperatures (heat and cold), the production of enzymes, amino acids, and B-group vitamins, which all are traits relevant in the dairy industry. Moreover, genes involved in the production of neurotransmitters serotonin and gamma-aminobutyric acid (GABA) were detected; however, *L. lactis* LL16 was able to produce only GABA during milk fermentation. The application of this strain contributes to the development of foods with improved functional qualities and higher food safety. Further in vivo studies are needed to evaluate the impact of *L. lactis* LL16 to gastrointestinal and neuroactive functions of the host.

## Data Availability

Data are contained within the article or Supplementary Material.

## Supplementary Materials

The following supporting information can be downloaded at: https://www.mdpi.com/article/10.3390/microorganisms11041034/s1, Figure S1: Closest phylogenetic relatives of *L. lactis* LL16 generated with RAST SEED analysis; Table S1: Probiotic characteristics based on *L. lactis* LL16 genome analysis with RAST SEED; Table S2: Annotation and localization (locus_tag) of the main features related to technological, functional and safety properties in *L. lactis* LL16 genome (blastKOALA).
