# Supplementary material for: Whole-Genome Sequence of Lactococcus lactis Subsp. lactis LL16 Confirms Safety, Probiotic Potential, and Reveals Functional Traits
Source: Microorganisms. 2023 Apr 15;11(4):1034. doi: 10.3390/microorganisms11041034 (PMC10145936; doi:10.3390/microorganisms11041034)
Supplement: Supplementary file 1 [file microorganisms-11-01034-s001.zip › microorganisms-2296019-supplementary.pdf]

Figure S1. Closest phylogenetic relatives of *L. lactis* LL16 generated with RAST SEED analysis

Genome ID	Score	Genome Name
272623.1	500	Lactococcus lactis subsp. lactis Il1403
889201.3	432	Streptococcus cristatus ATCC 51100
388919.8	432	Streptococcus sanguinis SK36
391296.7	430	Streptococcus suis 98HAH33
467705.8	430	Streptococcus gordonii str. Challis substr. CH1
205921.3	425	Streptococcus agalactiae A909
160491.17	422	Streptococcus pyogenes str. Manfredo
552526.7	421	Streptococcus equi subsp. zooepidemicus MGCS10565
160490.1	415	Streptococcus pyogenes M1 GAS
210007.1	405	Streptococcus mutans UA159
293653.3	404	Streptococcus pyogenes MGAS5005
171101.1	400	Streptococcus pneumoniae R6
299768.3	398	Streptococcus thermophilus CNRZ1066
246201.1	396	Streptococcus mitis NCTC 12261
226185.1	313	Enterococcus faecalis V583
565654.4	305	Enterococcus casseliflavus EC10
482234.3	196	Streptococcus canis FSL Z3-227
169963.1	193	Listeria monocytogenes EGD-e
525364.3	181	Lactobacillus salivarius ATCC 11741
272626.1	178	Listeria innocua Clip11262
563194.3	161	Pediococcus acidilactici 7_4
224308.1	135	Bacillus subtilis subsp. subtilis str. 168
321967.8	132	Lactobacillus casei ATCC 334
525328.4	129	Lactobacillus iners DSM 13335
387344.13	123	Lactobacillus brevis ATCC 367
272621.3	117	Lactobacillus acidophilus NCFM
491915.4	113	Anoxybacillus flavithermus WK1
279010.5	113	Bacillus licheniformis ATCC 14580
420246.5	110	Geobacillus thermodenitrificans NG80-2
235909.3	106	Geobacillus kaustophilus HTA426

Table S1. Probiotic characteristics based on *L. lactis* LL16 genome analysis with RAST SEED

Gene Function	Size (bp)
**Adhesion and aggregation**	
Enolase	1269
Fibronectin-binding protein	1623
Exopolysaccharides (EPS) biosynthesis	
EPS biosynthesis protein	840
Glycosyl transferase	1221/1329/321/1215/927
Glycosyl transferase, group 1 family protein	-
Glycosyl transferase, group 2 family protein	180/150/420/714/1056
Manganese-dependent protein-tyrosine phosphatase	420
Tyrosine-protein kinase EpsD	582
Tyrosine-protein kinase transmembrane modulator EpsC	684
Triosephosphate isomerase	759
AntiadhesinPls	-
Sortase A, LPXTG	1296/747
ATP synthase ε chain	426
DNA polymerase III, ε related 3’-5’ exonuclease	-
Mucus-binding protein	-
**Vitamin biosynthesis**	
**Thiamine**	
Cytosine/purine/uracil/thiamine/allantoin permease family protein	-
HMP-PP hydrolase (pyridoxal phosphatase) Cof	-
Hydroxyethylthiazole kinase	753
Hydroxymethylpyrimidine ABC transporter: ATPase component	-
Hydroxymethylpyrimidine ABC transporter: substrate-binding component	-
Hydroxymethylpyrimidine ABC transporter: transmembrane component	-
Hydroxymethylpyrimidine kinase	804
Thiamin ECF transporter: substrate-specific component ThiT	549
Thiazole ECF transporter: substrate-specific component ThiW	-
Thiamin pyrophosphokinase	636
Thiaminase II involved in salvage of thiamin pyrimidine moiety	657
Xanthine/uracil/thiamine/ascorbate permease family protein	1422
**Riboflavin**	
3,4-dihydroxy-2-butanone 4-phosphate synthase	-
6,7-dimethyl-8-ribityllumazine synthase	-
ATP phosphoribosyltransferase	-
Diacylglycerol kinase	-
Diaminohydroxyphosphoribosylaminopyrimidine deaminase/5-amino-6-(5-phosphoribosylamino)uracil reductase	-
Hypothetical protein YebC	-
NADH dehydrogenase	261/1260/2004
N-terminal domain of CinA protein, C-terminal domain of CinAtype S	-
Orotidine 5′-phosphate decarboxylase	-

Phosphoribosyl-AMP cyclohydrolase/Phosphoribosyl-ATP pyrophosphatase	639
Riboflavin kinase/FMN adenylyltransferase	-
Riboflavin synthase eubacterial/eukaryotic	-
Ribulose-phosphate 3-epimerase	651
Riboflavin ECF transporter: substrate-specific component RibU	-
Transcription termination protein NusB	858
tRNA pseudouridine synthase B	-
**Pyridoxin**	
D-3-phosphoglycerate dehydrogenase	1197
Hypothetical NagD-like phosphatase	774
1-deoxy-D-xylulose 5-phosphate synthase	-
Pyridoxamine 5′-phosphate oxidase	378/504
Pyridoxine biosynthesis glutamine amidotransferase, glutaminase subunit	-
NAD-dependent glyceraldehyde-3-phosphate dehydrogenase	1014/1011
**Biotin**	
3-ketoacyl-CoA thiolase/Acetyl-CoA acetyltransferase	696/276/144/1140
Acetoacetyl-CoA synthetase/Long-chain-fatty-acid–CoA ligase	-
Adenosylmethionine-8-amino-7-oxononanoate aminotransferase	-
Biotin ECF transporter: ATPase component BioM of energizing module	-
Biotin ECF transporter: substrate-specific component BioY	465/570
Biotin synthase	-
Biotin—protein ligase	753
Biotin operon repressor	972
Competence protein F homolog, phosphoribosyltransferase domain	651
Long-chain-fatty-acid–CoA ligase	1242
Biotin carboxyl carrier protein of acetyl-CoA carboxylase	468
Biotin carboxyl carrier protein of methylcrotonyl-CoA carboxylase	-
Biotin carboxylase of acetyl-CoA carboxylase	1368
Biotin carboxylase of methylcrotonyl-CoA carboxylase	-
**Folate**	
Dihydrofolate synthase/folylpolyglutamate synthase	1284
GTP cyclohydrolase I type 1	774
Pantoate—β-alanine ligase	-
Aspartate 1-decarboxylase	-
2-amino-4-hydroxy-6-hydroxymethyldihydropteridine pyrophosphokinase	1050
Dihydroneopterin aldolase	351
Dihydropteroate synthase	234/765
Cell division protein FtsH	2088
Hypoxanthine-guanine phosphoribosyltransferase	543/552
tRNA (Ile)-lysidine synthetase	1272
Para-aminobenzoate synthase, amidotransferase component	537
5-formyltetrahydrofolate cyclo-ligase	534
Dihydrofolate reductase	507

Thymidylate synthase	840
Dihydroneopterin triphosphate pyrophosphohydolase	498
**Amino acids metabolism**	
**Threonine**	
Biosynthetic aromatic amino acid aminotransferase α	-
Aspartokinase	1353
Homoserine dehydrogenase	1266
Aspartate aminotransferase	1131/1182/1155
Aspartate-semialdehyde dehydrogenase	1077
Homoserine kinase	849
Threonine synthase	1491
**Tryptophan**	
Tryptophan synthase α chain	762
Tryptophan synthase β chain	1209
Phosphoribosylformimino-5-aminoimidazole carboxamide ribotide isomerase	720
Para-aminobenzoate synthase, amidotransferase component	537
Isochorismatase	495/624
Indole-3-glycerol phosphate synthase	795
Anthranilate phosphoribosyltransferase	1008
Anthranilate synthase, amidotransferase component	597
Para-aminobenzoate synthase, aminase component (EC 2.6.1.85) / Aminodeoxychorismate lyase (EC 4.1.3.38)	1887
Isochorismate synthase (EC 5.4.4.2) Menaquinone-specific isochorismate synthase (EC 5.4.4.2)	474/606
Phosphoribosylanthranilate isomerase (EC 5.3.1.24)	1056
Anthranilate synthase, aminase component	1371
**Methionine**	
Cystathionine γ-lyase	1143
Cystathionine γ-synthase	1089
Cystathionine beta-lyase (EC 4.4.1.8)	603
S-adenosylmethionine synthase	123
Serine acetyltransferase	600
Homoserine O-succinyltransferase	960
Methionine ABC transporter ATP-binding protein	1107
5-methyltetrahydrofolate—homocysteine methyltransferase	1176/1059
5,10-methylenetetrahydrofolate reductase	852
O-acetylhomoserine sulfhydrylase	1281
SAM-dependent methlytransferase YrrT	-
**Leucine**	
3-isopropylmalate dehydrogenase	1038
Isopropylmalate isomerase	-
3-isopropylmalate dehydratase small subunit (EC 4.2.1.33)	576
3-isopropylmalate dehydratase large subunit (EC 4.2.1.33)	1383
2-isopropylmalate synthase	1542
**Lysine**	
Aspartokinase	1353
Lysine ε-oxidase	-
4-hydroxy-tetrahydrodipicolinate synthase	894

4-hydroxy-tetrahydrodipicolinate reductase	783
Aspartate-semialdehyde dehydrogenase	1077
Diaminopimelate decarboxylase	1260
N-acetyldiaminopimelate deacetylase	1155
2,3,4,5-tetrahydropyridine-2,6-dicarboxylate N-acetyltransferase	771
N-acetyl-L,L-diaminopimelate aminotransferase	1176
**Cysteine**	
Cysteine synthase	921/933
Phosphoadenylyl-sulfate reductase (thioredoxin)	315
Cysteinyl-tRNA synthetase	1347
Sulfite reductase [NADPH] hemoprotein β-component	-
Sulfite reductase [NADPH] flavoprotein α-component	-
**Histidine**	
Phosphoribosyl-AMP cyclohydrolase	639
Imidazole glycerol phosphate synthase, regulatory subunit	-
Imidazole glycerol phosphate synthase amidotransferase subunit HisH	609
Imidazole glycerol phosphate synthase cyclase subunit	780
Imidazole-glycerol-phosphate dehydratase	603
Histidinol dehydrogenase	114/1194
ATP phosphoribosyltransferase regulatory subunit (EC 2.4.2.17) HisGs	627/588/186
Histidinol-phosphate aminotransferase	555/537
Histidinol phosphatase	579/108
**Arginine**	
N-acetyl-γ-glutamyl-phosphate reductase	291/660
N-acetylglutamate synthase	1191
N-acetylglutamate kinase	852
N-acetylornithine aminotransferase	1134
Ornithine carbamoyltransferase	828/117
Argininosuccinate synthase	1197
Argininosuccinate lyase	1380
Arginine pathway regulatory protein ArgR	459
**Production of lactic acid**	
D-lactate dehydrogenase	153
L-lactate dehydrogenase	915/945/972/978
Predicted L-lactate dehydrogenase, Fe-S oxidoreductase subunit YkgE	771
Predicted L-lactate dehydrogenase, hypothetical protein subunit YkgG	684
Predicted L-lactate dehydrogenase, Iron-sulfur cluster-binding subunit YkgF	1473
**Active metabolism**	
Poly (glycerol-phosphate) α-glucosyltransferase	1452/1626
β-1,3-glucosyltransferase	900
Xylose isomerase domain protein TIM barrel	1320
**Enzyme production for food digestion**	
α-amylase	1578
Lipases	558

Serine protease extracellular	-
Serine protease, DegP/HtrA, do-like (EC 3.4.21.-)	1227
FIG056164: rhomboid family serine protease	693
Phytase	-
Cellulase	-
Xylanase	1134
**Stress adaptation / host gastrointestinal tract adaptation**	
**Temperature tolerance**	
Cold shock protein (CSP) family	132/159/201/159/123/168/ 150/198
Heat shock protein DnaJ	1140
Heat shock protein DnaK	1824
Heat shock protein HtpX	-
Heat shock protein Hsp33	-
Heat shock protein GrpE	540
Heat shock protein Hsp70	-
Small heat shock protein	-
Ribosome-associated heat shock protein	270
Co-chaperonin GroES (heat shock protein)	285
Molecular chaperone GroEL (heat shock protein)	1629
**Acid tolerance**	
ATP synthase subunit a	714
ATP synthase subunit b	507
ATP synthase subunit c	216
ATP synthase α chain	1503
ATP synthase β chain	1410
ATP synthase γ chain	870
ATP synthase δ chain	426
PTS system, cellobiose-specific IIC component	1338
ATP-dependent Clp protease ATP-binding subunit ClpB	1842/2604
ATP-dependent Clp protease ATP-binding subunit ClpC	2451
ATP-dependent Clp protease ATP-binding subunit ClpE	2247
ATP-dependent Clp protease ATP-binding subunit ClpP	600/702
ATP-dependent Clp protease ATP-binding subunit ClpX	666/543
Glucose-6-phosphate isomerase	1347
13 GTP pyrophosphokinase	561/681
Pyruvate kinase	1509
**pH tolerance**	
Sodium-proton antiporter	-
Alkaline shock proteins	402/369
F0F1-ATPase	-
**Bile salts tolerance**	
Choloylglycine hydrolase	-
Bile salt hydrolase	-
Glucosamine-6-phosphate deaminase	714/708
CTP synthase	1608
DamX	-
**Osmotic stress tolerance**	
Glycine betaine	-

ABC transporter, ATP-binding protein OpuAA	918
ABC transporter, ATP-binding protein BusAA	1227
ABC transporter, substrate binding protein and permease (BusAB.1/BusAB.2)	1638
ABC transporter, binding protein and permease (OpuAB/OpuAC)	1503
Manganese-dependent inorganic pyrophosphatase	-
**Oxidative stress tolerance**	
Catalase	-
Thiol peroxidase	483
Glutathione reductase	1308
Glutathione peroxidase	474
NADH peroxidase	-
NADH oxidase	1332/1341
NADH flavin oxidoreductase	-
Haloperoxidase	-
Iron dependent peroxidase	-
Thioredoxin reductase (NADPH)	966/927
Pyruvate oxidase	1704
Dihydroorotate oxidase	-
Cadmium-/manganese-transporting P-type ATPase	-
Manganese ABC transporter	762/714/906

Table S2. Annotation and localization (locus_tag) of the main features related to technological, functional and safety properties in *L. lactis* LL16 genome (blastKOALA).

Features Gene annotation	Gene	Locus_tag
**GAD gene cluster**		
Glutamate:gamma-aminobutyrate antiporter	gadC	INILNILJ_01034
Glutamate decarboxylase, gadB, gadA	gadAB	INILNILJ_01046
**Lactose utilisation**		
Lactose phosphotransferase system repressor	lacR	INILNILJ_01859
Galactose-6-phosphate isomerase subunit LacA	lacA	INILNILJ_01317
Galactose-6-phosphate isomerase subunit LacB	lacB	INILNILJ_01318
Tagatose-6-phosphate kinase	lacC	INILNILJ_01316
Tagatose 1,6-bisphosphate aldolase	lacD	INILNILJ_01315
PTS system, lactose-specific IIA component	lacF	INILNILJ_01314
PTS system, lactose-specific IIBC component	lacE	INILNILJ_01313
6-phospho-beta-galactosidase	lacG	INILNILJ_01312
Aldose 1-epimerase	lacX	INILNILJ_00669
**Proteolytic system**		
U32 family peptidase	prtC	INILNILJ_00675
**Oligopeptide permease system**		
Oligopeptide transport ATP-binding protein	OppD	INILNILJ_00002
Oligopeptide transport ATP-binding protein	OppF	INILNILJ_00001
Oligopeptide transport system permease protein	OppB	INILNILJ_00004
Oligopeptide transport system permease protein	OppC	INILNILJ_00003
Oligopeptide binding protein	OppA	INILNILJ_00005<br>INILNILJ_00006<br>INILNILJ_00239
